# Occurrence of erythema migrans in children with Lyme neuroborreliosis and the association with clinical characteristics and outcome – a prospective cohort study

**DOI:** 10.1186/s12887-018-1163-2

**Published:** 2018-06-11

**Authors:** Kesia Backman, Barbro H. Skogman

**Affiliations:** 1 School of Medical Sciences, Örebro University, S-702 81 Örebro, Sweden; 2 Pediatric Department, Falun County Hospital, Falun, Sweden; 3 Center for Clinical Research (CKF) Dalarna – Uppsala University, Nissers väg 3, S-791 82 Falun, Sweden

**Keywords:** Lyme neuroborreliosis, Erythema migrans, Facial nerve palsy, Clinical outcome, Children

## Abstract

**Background:**

Erythema migrans (EM) is the most common manifestation of Lyme borreliosis (LB), caused by the spirochete *Borrelia burgdorferi* sensu *lato*. The infection can disseminate into the nervous system and cause Lyme neuroborreliosis (LNB), the second most frequent LB manifestation in children. The aim of this prospective cohort study is to describe the occurrence of EM among children with LNB and to evaluate possible differences in clinical characteristics or outcome between LNB patients with and without EM.

**Method:**

Children being evaluated for LNB in southeast Sweden during the period 2010–2014 underwent a clinical examination, laboratory testing and filled out a questionnaire regarding duration and nature of symptoms, EM and the child’s health. Children were classified according to European guidelines for LNB. Clinical recovery was evaluated at a 2-month follow-up.

**Results:**

The occurrence of EM among children with LNB was 37 out of 103 (36%). Gender, age, observed tick bite, clinical features, duration of neurological symptoms or clinical outcome did not differ significantly between LNB patients with or without EM. However, facial nerve palsy was significantly more common among children with EM in the head and neck area.

**Conclusion:**

EM occurred in 36% of children with LNB and the location on the head and neck was more common among children with facial nerve palsy. EM was not associated with other specific clinical characteristics or outcome. Thus, the occurrence of EM in children with LNB cannot be useful as a prognostic factor for clinical outcome. This aspect has not previously been highlighted but seems to be relevant for the paediatrician in a clinical setting.

**Electronic supplementary material:**

The online version of this article (10.1186/s12887-018-1163-2) contains supplementary material, which is available to authorized users.

## Background

Lyme Borreliosis (LB) is the most common tick-borne infection in the Northern hemisphere [1, 2]. The spirochete *Borrelia burgdoferi* sensu *lato* is the etiologic agent for LB and in Europe, the most common genospecies causing human infection are *Borrelia (B) burgdoferi* sensu stricto *(s.s.)*, *B. garinii*, *B. afzelii* and in rare cases *B. spielmanii* [3–5].

Erythema migrans (EM) is the most frequent manifestation of LB in Europe and *B. afzelii* is the most common causative agent [3, 6]. EM is usually a ring-shaped expanding cutaneous lesion, localised at the origin of the tick-bite and with a clinical appearance pathognomonic for LB [1, 6]. In the early stage, the *Borrelia* infection can disseminate in to the bloodstream without causing generalised symptoms [7].

Lyme neuroborreliosis (LNB) is the second most frequent LB manifestation and most commonly caused by *B. garinii* [1–3]. The incidence of LNB in Sweden is 2.8/10.000 children [8]. The *Borrelia* spirochetes in the skin may spread into the central nervous system in two alternative ways: either through the bloodstream or along peripheral nerves [9]. Facial nerve palsy is the most commonly occurring clinical manifestation of LNB in children and it is often present at the ipsilateral side of the tick bite or EM [10]. Headache, fever and/or fatigue are common unspecific symptoms in LNB [8, 11] and occasionally, LNB presents with normal neurological examination [12]. Thus, the LNB diagnosis requires both clinical signs and symptoms attributable for LNB and laboratory testing, according to European guidelines [2, 13].

Clinical outcome after antibiotic treatment of EM is good [14], but persistent objective neurological signs and symptoms (persistent facial nerve palsy or other persistent motor/sensory deficits) after antibiotic treatment in paediatric LNB patients are reported in 11–27% of cases [11, 15]. Prognostic factors of importance for clinical recovery after LNB in childhood have not been found [11].

The aim of study is to describe the occurrence of EM among children with LNB, and to evaluate possible differences in clinical characteristics or outcome between LNB patients with and without EM.

## Methods

### Patients and controls

The study was performed at seven paediatric departments in a Lyme endemic area in southeast Sweden during the years 2010–2014. Children and parents/guardians were asked to participate in the study on admission and patients were enrolled in a prospective cohort. In total 306 patients were initially included in the study but a few patients (*n* = 11) were excluded due to missing clinical data or laboratory test results. Excluded patients (*n* = 11) did not differ in seasonal distribution, gender or age as compared to included patients (*n* = 295). Thus, patients in this present cohort study were considered representative of children being evaluated for LNB in a European Lyme endemic area. Patients were clinically examined by a paediatrician, underwent a lumbar puncture on admission and gave a blood sample for laboratory evaluation. A follow-up was conducted two months after admission, either as a visit to the paediatrician or as a telephone interview including a questionnaire for self-reported persistent symptoms.

### Classification of patients

Patients were classified as definite LNB, possible LNB, non-LNB or other specific diagnosis. The classifications of LNB patients were made according to European guidelines [13]. The three criteria for definite LNB were neurological signs and symptoms attributable to LNB without other obvious reason, pleocytosis in CSF and intrathecally produced anti-*Borrelia* antibodies (IgG and/or IgM). Possible LNB was defined as patients with two out of the three criteria above [13]. In this study, all possible LNB patient presented with neurological signs and symptoms attributable to LNB, pleocytosis in CSF, no intrathecally produced anti-*Borrelia* antibodies (IgG and/or IgM) and without clinical signs or laboratory evidence for other infection. Patients with definite LNB and possible LNB all received and responded well to antibiotic treatment and were thus considered as clinical LNB patients.

Patients who did not meet the criteria for definite LNB or possible LNB were classified as non-LNB or patients with other specific diagnoses.

EM was classified as an expanding round skin lesions, ≥5 cm in size [2], verified by a physician or self-reported.

### Laboratory evaluation

Pleocytosis was defined as total cell count > 5 × 10^6^ /L in CSF [16, 17]. Intrathecal anti-*Borrelia* antibody production (IgG and/or IgM) was analyzed with the routine assay IDEIA Lyme neuroborreliosis kit (Oxoid, Hampshire, UK) [18]. An index > 0.3 was considered as positive test for intrathecally produced anti-*Borrelia* antibodies according to manufacturer’s instructions. Data from anti-*Borrelia* antibodies in serum was not separately available for patients with positive index with the IDEIA assay, noted as NA (not available) for patients with definite LNB (Table 1).

**Table 1:** Clinical and laboratory characteristics of children in different diagnostic groups (*n* = 295)

On admission	Definite LNB (*n* = 68)	Possible LNB (*n* = 35)	Non-LNB (*n* = 133)	Other diagnosis (*n* = 59)
Gender				
Female, n (%)	30 (44)	15 (43)	83 (62)	30 (51)
Male, n (%)	38 (56)	20 (57)	50 (38)	29 (49)
Age, median (range)	6 (2–15)	8 (4–15)	13 (1–17)	10 (0–17)
Observed tick bite, n (%)	41 (60)	18 (51)	59 (44)	15 (25)
Clinical characteristics				
EM, n (%)	28 (41)	9 (26)	18 (14)	2 (3)
Facial nerve palsy, n (%)	46 (68)	25 (71)	51 (38)	5 (8)
Headache, n (%)	49 (72)	24 (69)	94 (71)	39 (66)
Fatigue, n (%)	62 (91)	23 (66)	88 (66)	38 (64)
Fever, n (%)	37 (54)	12 (34)	23 (17)	19 (32)
Neck pain, n (%)	36 (53)	18 (51)	35 (26)	19 (32)
Neck stiffness, n (%)	23 (34)	11 (31)	19 (14)	12 (20)
Loss of appetite, n (%)	43 (63)	19 (54)	46 (35)	19 (32)
Nausea, n (%)	24 (35)	12 (34)	46 (35)	23 (39)
Vertigo, n (%)	10 (15)	7 (20)	59 (44)	24 (41)
Laboratory findings				
Pleocytosis, median (range)	164 (20–890)	85 (6–1125)	0 (0–4)	0 (0–634)
Anti-*Borrelia* antibodies in CSF				
IgG, n (%)	18 (26)	0 (0)	0 (0)	0 (0)
IgM, n (%)	18 (26)	0 (0)	0 (0)	0 (0)
IgG + IgM, n (%)	32 (48)	0 (0)	0 (0)	0 (0)
Anti-*Borrelia* antibodies in serum				
IgG, n (%)	NA	0 (0)	0 (0)	0 (0)
IgM, n (%)	NA	1 (3)	0 (0)	0 (0)
IgG + IgM, n (%)	NA	19 (54)	16 (12)	1 (1)
Antibiotic treatment, n (%)	68 (100)	35 (100)	18 (11)	8 (14)

*EM* erythema migrans, *LNB* Lyme neuroborreliosis, *Ig* immunoglobulin, *NA* not available; pleocytosis = total cell count > 5 × 10^6^/L in CSF [17]; Anti-*Borrelia* antibodies in CSF are intrathecally produced and analyzed with the IDEIA assay [18]; patient are classified according to European guidelines [13]

### Antibiotic treatment

All patients diagnosed as clinical LNB were treated with antibiotics according to national guidelines; i.e. ceftriaxone i.v. 50–100 mg/kg once daily for 10–14 days for children < 8 years of age and doxycycline p.o. 4 mg/kg once daily for 10–14 days for children ≥8 years of age.

### Questionnaire

Patients (and/or parents/guardians) completed a structured questionnaire with questions about duration and nature of symptoms, observed tick bites, EM, lymphocytoma, previous treatment for LB and the child’s health on admission (Additional file 1). The paediatrician filled out a form with clinical information from the physical examination and the laboratory evaluation. At the 2-month follow-up, the patient (and/or parents/guardians) completed a structured questionnaire about characteristics and persistence of previously reported symptoms and the clinician evaluated the patient as recovered or not recovered. In some cases, medical records were scrutinized to obtain necessary information about clinical recovery.

### Statistics

Chi^2^ test and Fishers exact test were used for non-continuous data. For non-parametric analysis, the Mann-Whitney *U* test was used when comparing continuous data between groups. A *p*-value of < 0.05 was considered significant.

### Ethics

The study was approved by the Regional Ethics Committee in Uppsala, Sweden (Dnr 2010/106). Written informed consent was received from all parents/guardians.

## Results

Out of all 295 children being evaluated for LNB, 68 patients (23%) were classified as definite LNB, and 35 patients (12%) as possible LNB (Table 1). In total, 103 patients were categorised as clinical LNB patients and received antibiotic treatment. Non-LNB patients (*n* = 133) were mainly patients with idiopathic facial nerve palsy or headache of unknown origin, but a few children (*n* = 14) received antibiotic treatment due to uncertainties in laboratory diagnostics or a suspected EM (Table 1). Children with other specific diagnoses (*n* = 59) were patients diagnosed with tick-borne encephalitis (TBE), viral meningitis, post-infectious encephalitis, benign intracranial hypertension, epilepsy or various other neurological, immunological or infectious diseases. Some of these patients (*n* = 8) initially received treatment with antibiotics due to clinical suspicion of LNB, uncertainties in laboratory diagnostics or a suspected EM (Table 1). However, in those cases, antibiotic treatment was terminated when children were diagnosed as TBE (*n* = 2) or enteroviral meningitis (positive PCR in CSF) (*n* = 6) (data not shown).

Mononuclear cells were dominant (≥ 90%) in CSF pleocytosis in 67 out of 68 (99%) patients with definite LNB and 27 out of 35 (77%) patients with possible LNB. In patients with other specific diagnoses, pleocytosis occurred in 15 out of 59 (25%) patients. These were patients with tick-borne encephalitis (TBE), viral meningitis or post-infectious encephalitis and 10 out of 15 had ≤90% mononuclear cells in CSF pleocytosis (data not shown).

Clinical and laboratory characteristics of the patients being evaluated for LNB and controls are shown more in detail in Table 1. Patients with definite LNB or possible LNB were younger than patients in Non-LNB or other diagnosis (Table 1). Facial nerve palsy, headache and fatigue were common symptoms among children with LNB but also among controls. Observed tick bites and/or EM occurred in all diagnostic groups but most frequently in definite LNB (Table 1).

**Fig. 1:**
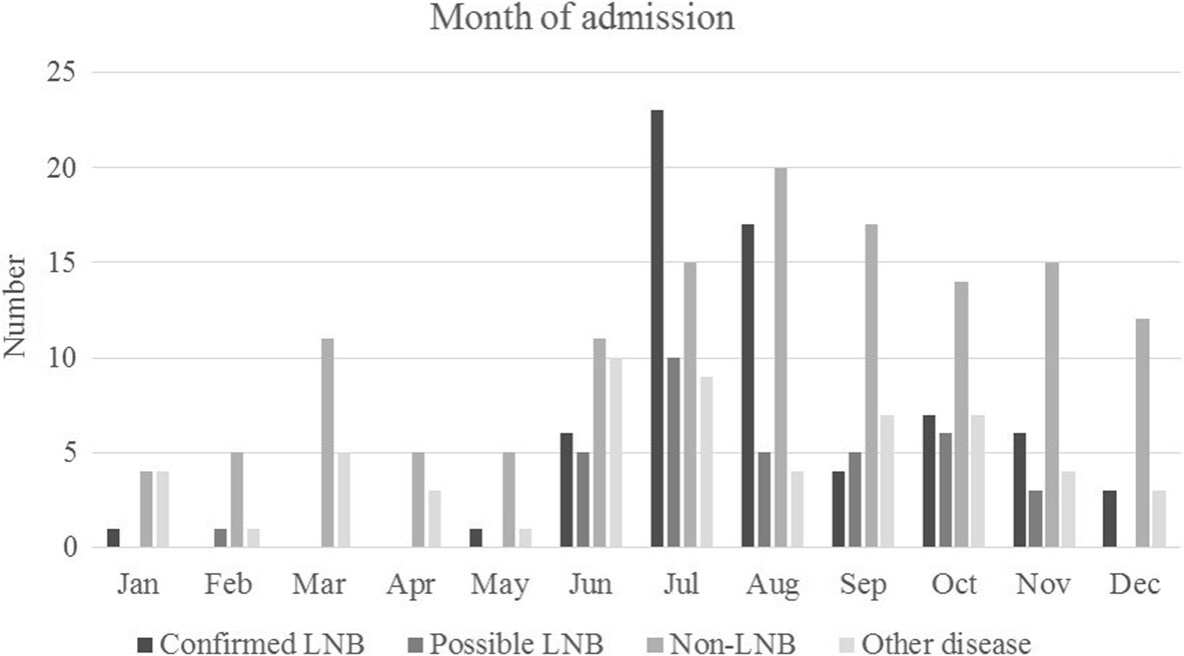
Month of admission for patients (*n* = 295) being evaluated for Lyme neuroborreliosis (LNB)

Children were evaluated for LNB throughout the whole year, but with a higher incidence of LNB cases during June–December (Fig. 1). The one patient diagnosed with definite LNB in January reported a tick bite 1–2 months before, and the duration of neurological symptoms was 3–6 days. One patient was diagnosed with possible LNB in February; this patient had symptoms such as headache, loss of appetite, vertigo and radiant pain in limbs since more than 2 months. She had a lymphocytoma on the left earlobe and had observed a tick-bite 6–12 months earlier. She had pleocytosis in CSF but no intrathecally produced anti-*Borrelia* antibodies.

**Fig. 2:**
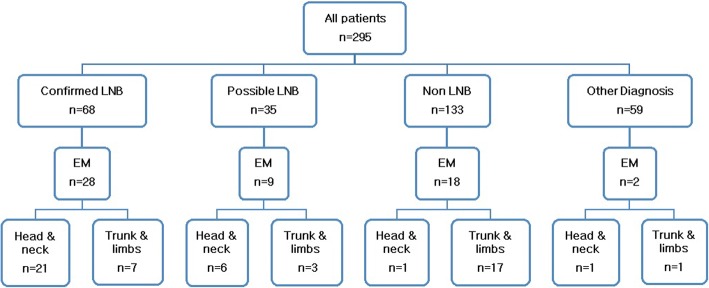
Occurrence and location of erythema migrans (EM) among patients with Lyme neuroborreliosis (LNB) and controls

EM occurred in a total of 57 patients and was seen in all four diagnostic groups (Fig. 2). The most common location of the EM was the head and neck area (*n* = 29) (Fig. 2, Table 2). There were no reports of multiple EM. Facial nerve palsy was significantly more common in patients with EM in the head and neck area as compared to patients with EM on the trunk and limbs (Table 2). Children with EM in the head and neck area were younger (median age 6 years) compared to children with EM on the trunk and limbs (median age of 10 years) (*p* < 0.01).

**Table 2:** Location of erythema migrans compared to the occurrence of facial nerve palsy in all patients with erythema migrans (*n* = 57)

	EM head & neck (*n* = 29)	EM trunk & limbs (*n* = 22)	*p*-value
Facial nerve palsy			
Yes, n (%)	20 (69)	5 (23)	0.002
No, n (%)	9 (31)	17 (77)	

*EM* erythema migrans

The characteristics of patients diagnosed with LNB and EM are shown separately in Table 3. Most patients had a short time duration between EM and LNB diagnosis and the lesion was often located in the head and neck area (Table 3). Out of all LNB patients with EM (*n* = 37), only four patients (11%) had previously received antibiotic treatment for the EM. They had been treated with phenoxymethyl penicillin (*n* = 3) and amoxicillin (*n* = 1) a few weeks earlier (1–4 weeks). Three of these children were definite LNB patients with pleocytosis and intrathecally produced anti-*Borrelia* antibodies and one patient was classified as possible LNB with pleocytosis but no intrathecally produced anti-*Borrelia* antibodies. All four patients were fully recovered at the 2-months follow-up. In the Non-LNB group, one of the patients with idiopathic facial nerve palsy had EM but no IgM serum antibodies. Unfortunately, no follow-up serology was performed, so the diagnosis may be uncertain. He was fully recovered at the clinical follow-up.

**Table 3:** Clinical characteristics of patients with Lyme neuroborreliosis and erythema migrans

On admission	LNB patients with EM (*n* = 37)
Gender	
Female, n (%)	14 (38)
Male, n (%)	23 (62)
Age, median (range)	7 (2–15)
Observed tick bite, n (%)	25 (68)
Time between EM and LNB diagnosis	
1–4 weeks, n (%)	11 (30)
1–2 months, n (%)	9 (24)
3–5 months, n (%)	0 (0)
6–12 months, n (%)	0 (0)
> 1 year, n (%)	1 (3)
Not specified, n (%)	16 (43)
Location of EM	
Head and neck, n (%)	27 (73)
Trunk, n (%)	2 (5)
Limbs, n (%)	6 (16)
Not specified, n (%)	2 (5)
Antibiotic treatment for EM	4 (11)

*EM* erythema migrans, *LNB* Lyme neuroborreliosis; patient are classified according to European guidelines [13]

No significant differences in clinical characteristics on admission were found when comparing LNB patients with and without EM (Table 4). LNB patients with EM had the same clinical outcome as LNB patients without EM, and there were no significant differences in character or frequency of the persistent symptoms between the two groups (Table 4).

**Table 4:** Clinical characteristics and comparison between Lyme neuroborreliosis patients with or without erythema migrans

On admission and at follow-up	Patients with LNB and EM (*n* = 37)	Patients with LNB without EM (*n* = 66)	*p*-value
Gender			
Female, n (%)	14 (38)	31 (47)	
Male, n (%)	23 (62)	35 (53)	0.37
Age, median (range)	7 (2–15)	7 (2–15)	0.53
Observed tick bite, n (%)	25 (68)	34(52)	0.11
Clinical characteristics			
Facial nerve palsy, n (%)	26 (70)	45 (68)	0.83
Headache, n (%)	23 (62)	50 (76)	0.15
Fatigue, n (%)	34 (92)	51 (77)	0.10
Fever, n (%)	21 (57)	28 (42)	0.16
Neck pain, n (%)	17 (46)	37 (56)	0.32
Neck stiffness, n (%)	13 (35)	21 (32)	0.73
Loss of appetite, n (%)	22 (60)	40 (61)	0.91
Nausea, n (%)	13 (35)	23 (35)	0.98
Vertigo, n (%)	7 (19)	10 (15)	0.62
Duration of neurological symptoms			
1–2 days, n (%)	5 (14)	6 (9)	0.52
3–6 days, n (%)	17 (46)	25 (38)	0.42
1–2 weeks, n (%)	7 (19)	19 (29)	0.27
2–4 weeks, n (%)	5 (14)	10 (15)	1.00
1–2 months, n (%)	1 (3)	0 (0)	0.36
> 2 months	1 (3)	2 (3)	1.00
Not specified, n (%)	1 (3)	4 (6)	0.65
Clinical outcome			
Total recovery within 2 months, n (%)	31 (84)	56 (85)	0.43
Major persistent symptom			
Facial nerve palsy, n (%)	2 (5)	7 (11)	0.48
Headache, n (%)	1 (3)	2 (3)	1.00
Fatigue, n (%)	1 (3)	0 (0)	0.36

*EM* erythema migrans, *LNB* Lyme neuroborreliosis; patient are classified according to European guidelines [13]

## Discussion

In this present study, the occurrence of EM was 36% among children with clinical LNB, which is similar to previous studies from Europe where LNB patients presented with or reported previous EM in 23–31% of cases [11, 16, 19, 20]. Sex, age, observed tick bite, clinical characteristics and duration of neurological symptoms did not differ significantly between LNB patients with and without EM in our study. However, among children with LNB and EM in the head and neck area, the occurrence of facial nerve palsy was significantly higher. This is in line with previous studies, supporting the hypothesis that spirochetes can disseminate through the skin into the cranial nerve and the central nervous system [1, 10]. In a study of paediatric patients with LNB, children with EM in the head and neck area presented with ipsilateral facial nerve palsy in 94% of cases [10].

LNB patients presenting with EM in the head and neck area were younger compared to patients with EM on trunk or limbs in our study. This could possibly be explained by the fact that younger children are shorter and move in nature in a way that they receive tick-bites more easily in the head and neck area.

Clinical outcome did not differ between LNB patients with and without EM in our present study, nor in total recovery rate or in character or frequency of persistent symptoms. In previous studies on children with LNB [3, 11, 20], the aspect of comparing outcome in LNB patients with and without EM has not been focused upon, which makes our findings interesting. Thus, the occurrence of EM in paediatric LNB patients does not seem to be a prognostic factor for clinical outcome.

The majority of LNB patients with EM (89%) had not received antibiotic treatment for their EM prior to the LNB diagnosis. Thus, most patients who developed LNB were untreated in our study and the knowledge regarding EM seems to have been low. On the other hand, four patients (11%) had received antibiotic treatment for EM according to guidelines (i.e. phenoxymethyl penicillin p.o. 25 mg/kg × 3 for 10 days) but still developed LNB. This is of course unsatisfactory but may be explained by the fact that some spirochetes could have disseminated rapidly from the skin into the central nervous system before penicillin had had the chance to eradicate the spirochetes at the site of the skin infection.

Of all our LNB patients with EM, 62% were male. The male predominance is consistent with previous studies on LNB patients [11, 15, 20, 21]. Gender differences have been described in a previous study concerning distribution of acute facial nerve palsy, headache and neck stiffness among children with LNB [22]. However, that study showed no significant gender differences concerning the occurrence of EM among children with LNB, which is congruent with our results.

A strength of this study is that it is a representative cohort of patients evaluated for LNB in a large European Lyme endemic area. Additionally, the study was conducted over several years, which avoided bias connected to yearly variations in tick abundancy or incidence of LNB in the population.

Since EM was not found to be a prognostic factor for clinical outcome in LNB, it could have been of interest with some information about the *Borrelia* genospecies causing LNB, in this material. *B.afzelii* usually causes skin lesions and *B.garinii* causes LNB [23]. Unfortunately, PCR analyses in CSF for detection and sequencing of DNA from *Borrelia* genospecies were not performed on admission in the majority of cases in this study. However, CSF was analysed in a few LNB patients (*n* = 6) where *B.garinii* was detected in 3 cases, *B.afzelii* in one patient, *B.bavarensis* in one patient and an unspecified genospieces in one patient (unpublished data). The clinical characteristics of these few patients did not differ apparently, but the data are not suitable for further analysis and the question of whether genospecies has prognostic importance for LNB patients with or without EM cannot be answered here.

## Conclusion

EM occurred in 36% of children with LNB and the location in the head and neck area was more common among children with facial nerve palsy. However, EM was not associated with other specific characteristics or clinical outcome. Thus, the occurrence of EM in children with LNB can not be useful as a prognostic factor for clinical outcome. This aspect has not previously been highlighted, but seems to be relevant for the paediatrician in a clinical setting.

## Additional file

Additional file 1: Questionnaire. A structured questionnaire with questions about duration and nature of symptoms, observed tick bites, EM, lymphocytoma, previous treatment for LB and the child’s health on admission. (PDF 138 kb)

## Abbreviations

CSF: Cerebrospinal fluid
DNA: Deoxyribonucleic acid
EM: Erythema migrans
Ig: Immunoglobulin
LB: Lyme borreliosis/Lyme disease
LNB: Lyme neuroborreliosis
PCR: Polymerase chain reaction
TBE: Tick-borne encephalitis
