# Supplementary material for: Occurrence of erythema migrans in children with Lyme neuroborreliosis and the association with clinical characteristics and outcome – a prospective cohort study
Source: BMC Pediatr. 2018 Jun 11;18:189. doi: 10.1186/s12887-018-1163-2 (PMC5996539; doi:10.1186/s12887-018-1163-2)
Supplement: Supplementary file 1 — Questionnaire. A structured questionnaire with questions about duration and nature of symptoms, observed tick bites, EM, lymphocytoma, previous treatment for LB and the child’s health on admission. (PDF 138 kb) [file 12887_2018_1163_MOESM1_ESM.pdf]

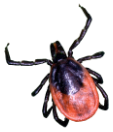

Study ”Lyme Neuroborreliosis in children”

Questionnaire (by parents and/or guardians) Code:________________________

Name:____Hospital:______________________

Date of birth: ____________________________________Date for inclusion:______________

****

Questions:

	YES	NO	If yes, duration of symptoms at admission: 1-2 d	3-6 d	1-2 w	2-4 w	1-2 m	>2 m
1. Facial nerve palsy:	☐	☐	☐	☐	☐	☐	☐	☐
2. Headache:	☐	☐	☐	☐	☐	☐	☐	☐
3. Fatigue:	☐	☐	☐	☐	☐	☐	☐	☐
4. Fever (38-39^0 celcius):	☐	☐	☐	☐	☐	☐	☐	☐
5. Neck pain:	☐	☐	☐	☐	☐	☐	☐	☐
6. Neck stiffness:	☐	☐	☐	☐	☐	☐	☐	☐
7. Loss of appetite:	☐	☐	☐	☐	☐	☐	☐	☐
8. Nausea/vomiting:	☐	☐	☐	☐	☐	☐	☐	☐
9. Vertigo:	☐	☐	☐	☐	☐	☐	☐	☐
10. Radiating pain	☐	☐	☐	☐	☐	☐	☐	☐
11. Other symptom:	☐	☐	☐	☐	☐	☐	☐	☐

(if yes, what symptom:..
....)

	YES	NO	If yes, duration at admission: 1-4 w	1-2 m	3-5 m	6-12 m	>1 y
12. Tick bite: (if yes, where?...)	☐	☐	☐	☐	☐	☐	☐
13. Red skin lesion (erythema migrans): (if yes, where?...)	☐	☐	☐	☐	☐	☐	☐
14. Swollen earlobe (lymphocytoma):	☐	☐	☐	☐	☐	☐	☐
15. Vesicles on lip (herpes simplex):	☐	☐	☐	☐	☐	☐	☐
16. Vesicles on skin (varicella zoster):	☐	☐	☐	☐	☐	☐	☐
17. Vaccination for TBE:	☐	☐	☐	☐	☐	☐	☐
18. Vaccination for Yellow fever:	☐	☐	☐	☐	☐	☐	☐

****

19. Treatment for previous *Borrelia* infection (drug, dose)?……………………………………..
………………………………………………………………………………………………

20. Where in Sweden has the child been spending summer vacation the last two years?
………………………………………………………………………………………………

21. Is the child healthy (yes/no)?...

22. If no, what is the problem (diagnosis)?...
....

23. Treatment?..
